# Basolateral amygdala rapid glutamate release encodes an outcome-specific representation vital for reward-predictive cues to selectively invigorate reward-seeking actions

**DOI:** 10.1038/srep12511

**Published:** 2015-07-27

**Authors:** Melissa Malvaez, Venuz Y. Greenfield, Alice S. Wang, Allison M. Yorita, Lili Feng, Kay E. Linker, Harold G. Monbouquette, Kate M. Wassum

**Affiliations:** 1 Dept. of Psychology, UCLA, Los Angeles, CA 90095, USA; 2 Dept. of Chemical Engineering, UCLA, Los Angeles, CA 90095, USA; 3 Brain Research Institute, UCLA, Los Angeles, CA 90095, USA

## Abstract

Environmental stimuli have the ability to generate specific representations of the rewards they predict and in so doing alter the selection and performance of reward-seeking actions. The basolateral amygdala participates in this process, but precisely how is unknown. To rectify this, we monitored, in near-real time, basolateral amygdala glutamate concentration changes during a test of the ability of reward-predictive cues to influence reward-seeking actions (Pavlovian-instrumental transfer). Glutamate concentration was found to be transiently elevated around instrumental reward seeking. During the Pavlovian-instrumental transfer test these glutamate transients were time-locked to and correlated with only those actions invigorated by outcome-specific motivational information provided by the reward-predictive stimulus (*i.e.*, actions earning the same specific outcome as predicted by the presented CS). In addition, basolateral amygdala AMPA, but not NMDA glutamate receptor inactivation abolished the selective excitatory influence of reward-predictive cues over reward seeking. These data the hypothesis that transient glutamate release in the BLA can encode the outcome-specific motivational information provided by reward-predictive stimuli.

Adaptive reward seeking is critical to survival and is disrupted in a variety of neuropsychiatric disorders, including substance abuse, overeating and depression. The basolateral amygdala (BLA) has been implicated in these disorders^1^,^2^,^3^,^4^ and is involved in reward processing^5^,^6^, but much is unknown about its precise contribution. The BLA receives dense cortical and thalamic glutamatergic input^7^,^8^,^9^. Based on the results of BLA lesions^10^,^11^,^12^,^13^, these excitatory chemical messages may be thought to convey a sustained emotional valence signal in response to reward-predictive stimuli. However, it is also possible that BLA signaling represents the motivational value of specific reward expectations generated by such cues. Here we investigate the latter.

Assessment of this hypothesis requires a method to selectively measure BLA glutamate signaling with fast temporal resolution in order to distinguish chemical messages related to individual reward-seeking behaviors. Microdialysis allows for selective measurement of extracellular neurochemical concentration changes, but the typical 10–20 min (or even rapid 14–20 s^14^,^15^,^16^) sampling window does not provide the appropriate temporal resolution and the spatial resolution is inadequate to record from BLA microenvironments. Single-unit electrophysiological recordings provide the required temporal and spatial resolution, but are non-selective and biased to record mostly from output neurons, precluding evaluation of glutamatergic input or local processing of such chemical messages within the BLA. Biosensor technologies^17^,^18^,^19^,^20^ provide a solution. Using an electroenzymatic approach, this technique allows online, near-real time, sensitive and selective measurement of extracellular glutamate concentration changes that result from neuronal release^21^,^22^,^23^,^24^,^25^. Recent biosensor data support the possibility that BLA glutamate release *may* convey information important for reward seeking; transient fluctuations in extracellular BLA glutamate concentration were detected immediately preceding reward-seeking actions^21^, but the precise information encoded by these glutamate transients is unknown.

One major source of reward-seeking motivation is the cognitive expectation of specific available rewards, information that is often provided by environmental stimuli. Indeed, an environmental reward-predictive stimulus will selectively invigorate the performance of those actions that earn the same specific reward associated with the stimulus^26^,^27^,^28^,^29^. This capacity requires the BLA^11^,^13^,^30^ and is thought to rely upon retrieval of a cognitive representation of the specific shared reward (*i.e.*, outcome) encoded in both the Pavlovian stimulus-outcome and instrumental action-outcome association^31^,^32^. BLA neurons can fire in response to reward-predictive cues^33^,^34^,^35^,^36^,^37^ and in anticipation of reward^38^, but the chemical message driving this neuronal activity and whether it encodes the motivational value of specific reward representations has yet to be clarified.

Therefore, we evaluated the role of BLA glutamate signaling in outcome-specific Pavlovian-instrumental transfer (PIT). In this task, rats are trained to associate two auditory Pavlovian stimuli (CS) with two distinct food rewards and then to respond on two independent levers to earn the same rewards. In the critical PIT test both levers are available and the CS will selectively enhance the response with which it shares a rewarding outcome^26^,^27^,^28^,^32^. Because the CSs are never directly associated with the instrumental actions, this test assesses the rats’ ability to mentally represent each specific reward and to use this outcome-specific information to guide and motivate reward seeking. We reasoned that if BLA glutamate release is related to the motivational influence of cue-induced, outcome-specific representations, then blocking ionotropic glutamate receptors (iGluRs) should disrupt the selective excitatory influence of the cue on reward seeking and, under normal conditions, glutamate release should precede and correlate with only those actions that are selectively invigorated by the CS.

## Results

### Experiment 1

**Figure 1:**
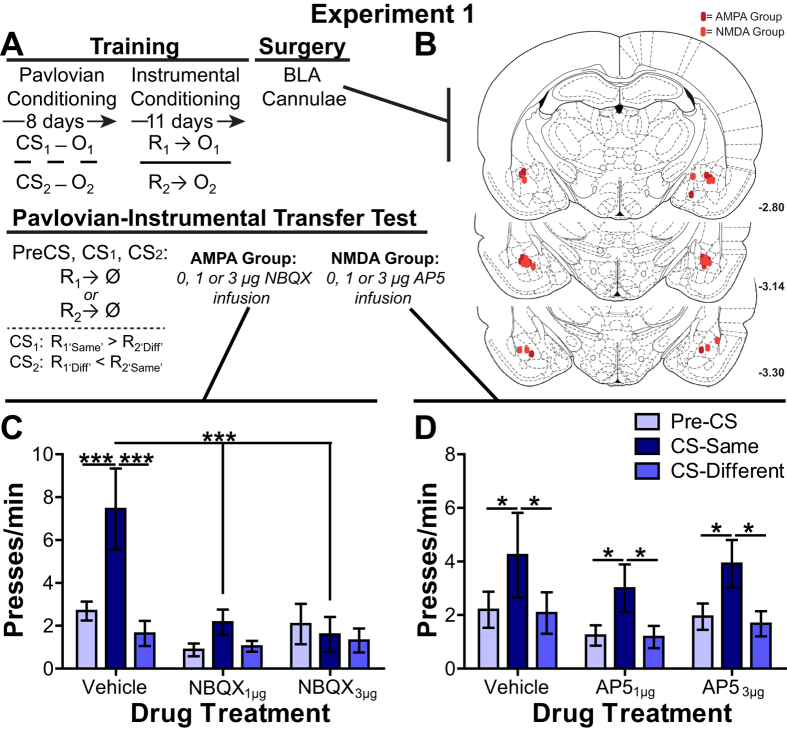
Effect of basolateral amygdala AMPA or NMDA receptor inactivation on Pavlovian-instrumental transfer. (**A**) Experimental procedure (see methods). CS, conditioned stimulus; O, outcome/reward; R, instrumental lever-press response; Ø, no reward delivery. During the PIT test both levers were available, but pressing was not reinforced and each CS was presented 4 times with intervening no-cue (Pre-CS) periods serving as a control. During CS presentation actions on the lever that, during training, earned the same outcome as the cue predicted were considered ‘same’ presses, while actions on the other available lever were considered ‘different’ (Diff) presses. (**B**) Schematic representation of microinfusion injector tips. Numbers to the lower right of each section represent the anterior-posterior distance (mm) from bregma of the section. Line drawings of coronal sections are taken from^73^. (**C**,**D**) The Pavlovian-instrumental transfer effect; Lever-press rate (presses/min) averaged across levers during the control Pre-CS periods compared to pressing on lever that, in training, earned the same outcome as predicted by the CS (CS-Same) relative to pressing on the opposite lever (CS-Different) for the AMPA antagonist (**C**) or NMDA antagonist (**D**) group. Error bars +1 SEM. *p < 0.05, **p < 0.01, ***p < 0.001.

In Experiment 1 (see Fig. 1A) we pharmacologically blocked either BLA AMPA (0, 1 or 3 μg/side of NBQX) or NMDA (0, 1 or 3 μg/side of AP5) glutamate receptors prior to the outcome-specific PIT test in order to assess their respective contributions to the selective invigorating influence of reward-predictive cues. During the PIT test both levers were simultaneously present, but pressing was never rewarded. Each CS was presented 4 times in alternating order, with intervening control CS-free periods (pre-CS). In this test the CS presentation provides the cognitive information (*e.g.*, specific reward representation) that guides action selection and performance in the novel choice scenario.

#### BLA AMPA and NMDA glutamate receptor involvement in the selective invigorating influence of reward-predictive stimuli over reward-seeking actions

As is clear from Fig. 1C,D, we detected a differential effect of AMPA and NMDA iGluR receptor blockade on the selective-invigorating influence of reward-predictive stimuli over reward seeking during the outcome-specific PIT test. For the AMPA group there was a significant main effect of both CS (F_2,14_ = 12.06, *p* < 0.001) and NBQX dose (F_2,14_ = 4.92, *p *=* *0.02) on lever pressing, as well as a significant interaction between these factors (F_4,28_ = 5.26, *p *=* *0.003). Following a control vehicle infusion CS presentation selectively elevated press rate on the lever that, in training, earned the same reward as predicted by the CS (CS-Same) relative to both pre-CS press rate (*p* < 0.001) and pressing during the CS on the alternate available lever (CS-Different; *p* < 0.001). This selective elevation on the CS-Same action was blocked by BLA AMPA receptor inactivation (*p *>* *0.05, for both doses) and, indeed, CS-Same responding was lower following intra-BLA NBQX infusion than vehicle control (*p* < 0.001, for both doses). Intra-BLA NBQX did not significantly alter pre-CS baseline or CS-Different response rates (*p *>* *0.05), suggesting a specific effect of AMPA receptor blockade on the selective invigorating influence of cues over action performance. The low response rate during the pre-CS period may have, however, been close to the floor for detecting a significant decrease in responding. To ensure AMPA receptor blockade did not alter baseline responding we isolated the first PIT trial for which the pre-CS response rate was higher (~5 presses/min) and in this case found identical results to the trial-averaged data; AMPA receptor blockade selectively attenuated CS-Same responding and did not significantly impact pre-CS response rate (see Supplemental Fig. 1).

Blockade of BLA NMDA receptors was without effect on outcome-specific PIT (Fig. 1D). For the NMDA group there was a main effect of CS (F_2,16_ = 18.68, *p* < 0.001), with neither an effect of AP5 dose (F_2,16_ = 0.46, *p *=* *0.64), nor AP5 dose x CS interaction (F_4,32_ = 0.04, *p *=* *0.99). Under each drug dose treatment the CS elevated responding on the CS-Same action relative to both the pre-CS period and to the CS-Different action (*p* < 0.05, in all cases). After both intra-BLA AMPA and NMDA receptor blockade rats were able to show a CS-induced elevation in Pavlovian conditioned food-port approach responding (see Supplemental Results and Supplemental Fig. 2).

### Experiment 2

**Figure 2:**
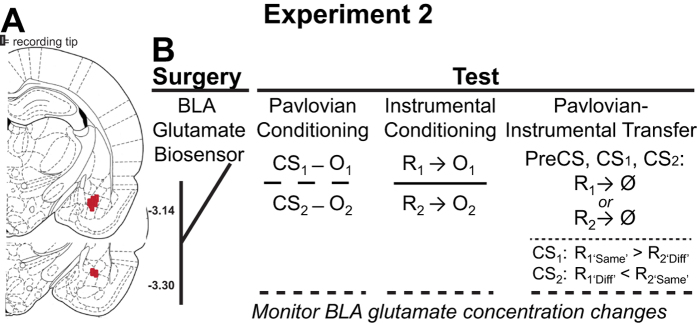
Experiment 2 Design. (**A**) Schematic representation of the placement of the microelectrode array biosensor tips. Numbers to the lower right of each section represent the anterior-posterior distance (mm) from bregma of the section. (**B**) Testing procedures (see methods). CS, conditioned stimulus; O, outcome/reward; R, instrumental lever-press response; Ø, no reward delivery. During the PIT test both levers were available, but pressing was not reinforced and each CS was presented 4 times with intervening no-cue (Pre-CS) periods. During the CS presentation actions on the lever that, during training, earned the same outcome as the cue predicted were considered ‘same’ presses, while actions on the other lever were considered ‘different’ (Diff) presses. BLA glutamate concentration changes were continuously monitored with constant potential amperometry at glutamate biosensors during each test.

The results of Experiment 1 suggest that BLA AMPA iGluR activation is necessary for reward-paired cues to selectively invigorate the performance of a specific reward-seeking action. We next used electroenzymatic biosensors to make sub-second measurements of extracellular glutamate concentration changes to interrogate the profile of BLA glutamate release during instrumental conditioning and PIT (see Fig. 2B). We reasoned that if BLA glutamate signaling is related to the motivational value of reward-specific representations, then such signaling might correlate with the performance of reward-seeking actions during instrumental conditioning. More importantly, during the critical PIT test BLA glutamate signaling should correlate with the performance of only those actions that are selectively motivated by the CS-generated reward representation (*i.e.*, CS-Same pressing).

#### BLA glutamate release during instrumental conditioning

**Figure 3:**
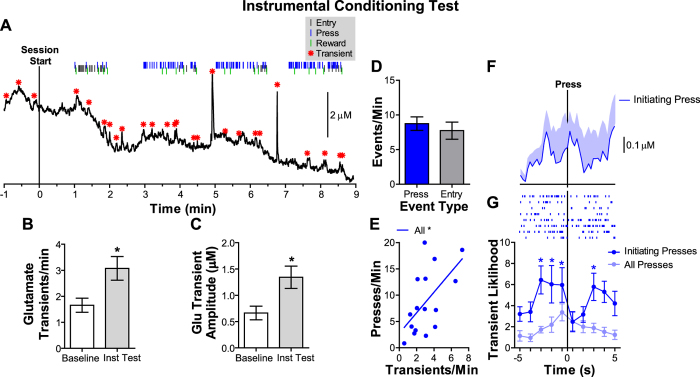
Basolateral amygdala glutamate release during instrumental conditioning. (**A**) Representative glutamate concentration v. time trace during instrumental conditioning. Instrumental session started with lever insertion at time 0 s. Red asterisks represent significant transient glutamate concentration fluctuations above baseline (transients). Behavioral events are marked above the trace. (**B**) BLA glutamate transients that reached threshold were counted and averaged for each rat across the 2-min pre-test baseline period and the instrumental (inst) test sessions. (**C**) Glutamate transient amplitude (μM) was calculated as the peak amplitude of the transient minus the baseline concentration (first minima 0.5–5 s before the peak). (**D**) Lever press and food-port entry rate collapsed across the instrumental conditioning tests. (**E**) Between-subject correlation between glutamate transient frequency (Transients/min) and lever-press rate (Presses/min). Each rat is represented twice, once for each instrumental test. (**F**) Glutamate concentration v. time trace around initiating lever presses (occurring at time 0 s) averaged across all initiating presses in the session for a representative subject. Shading reflects +1 SEM across trials. (**G**) The likelihood of a glutamate transient in 10, 1-s bins, evenly distributed around presses divided by initiating presses (first press after the collection of an earned reward or after a >6 s pause in pressing, excluding presses that occurred within a pressing bout) or all lever presses combined (including both initiating and intra-bout presses), averaged across the two instrumental tests. The press occurs at time 0 s. Likelihood of a glutamate transient is defined as the percentage of presses (or initiating presses) that had a glutamate transient in the represented 1-s time bin. Asterisks represent significance relative to the control 1-s time bin, 5 s prior to the press. The raster plot shows the corresponding raw data with each subject represented on an individual line on the y-axis, tick marks represent the peak time of each significant glutamate transient surrounding initiating presses. Error bars +1 SEM. *p < 0.05, ***p < 0.001.

As can be seen in the representative example presented in Fig. 3A, during the instrumental reward-seeking test there were rapid, short-duration increases in glutamate concentration (*i.e.*, glutamate transients) that were increased in both frequency (Fig. 3B; t_7_ = 2.34, *p *=* *0.05) and amplitude (Fig. 3C; t_7_ = 2.85, *p *=* *0.02) during instrumental performance, relative to the pre-test baseline period. See Supplemental Figure 3 for further details on transient amplitude. Interestingly, the frequency of these glutamate release events positively correlated (r_16_ = 0.58, *p *=* *0.02; Fig. 3E) with lever-press rate (see Fig. 3D), such that higher press rates were associated with more frequent BLA glutamate transients.

As can also be seen in Fig. 3A, the frequency of glutamate transients fluctuated throughout the instrumental conditioning test and appeared to share a tight temporal relationship to lever-press actions, especially those actions initiating bouts of reward seeking (see representative trial-averaged glutamate concentration v. time trace in Fig. 3F). To specifically evaluate the relationship between glutamate release events and instrumental reward seeking we calculated the likelihood of a glutamate transient in the time immediately surrounding lever presses. Because rats tended to organize their lever pressing into clusters we divided our analysis for those presses that initiated reward seeking (*i.e*., ‘initiating presses’) excluding presses that occurred within a pressing bout and compared this to all lever presses (including both initiating and intra-bout presses). Initiating presses were defined as the first press after collection of an earned reward or the first press after a >6 s pause in pressing. During the instrumental test rats showed on average 34.25 (sem = 5.53) total reward-seeking bouts per session, with 23.68% (1.77) of total presses being considered ‘initiating presses’. Reward-seeking bouts had an average duration of 8.20 s (1.33), and contained on average 5.84 (1.03) presses. The average reward receipt to next initiating press latency was 23.10 s (7.47). To isolate the initiation of instrumental reward seeking and avoid the presence of contaminating events (*e.g*., reward receipt, termination of previous bout, etc.) in the reward-seeking initiation analysis window we calculated the change in likelihood of a glutamate transient by counting the glutamate transients in 10, 1-s bins evenly distributed around presses. A longer analysis window are presented in the Supplemental Results and Supplemental Figures 4.

The raw glutamate transient counts around initiating presses for each subject are displayed in the raster plot shown in Fig. 3G. Statistical analysis of the data collapsed over 1-s intervals (to match biosensor response time) and averaged across subjects (Fig. 3G- bottom) found a marginally insignificant effect of Time surrounding the press (F_9,63_ = 1.79, *p *=* *0.08), a significant effect of Type of press (Initiating press v. All presses, F_1,7_ = 61.42, *p *=* *0.0001) and a Time x Type of press interaction (F_9,63_ = 2.67, *p *=* *0.01; Fig. 3G). Glutamate transients were more likely (when controlling for number of presses) to occur time-locked to those initiating presses that followed either reward delivery or a pause in pressing than all presses combined. The likelihood of a glutamate transient was elevated (relative to the control 1-s time bin, 5 s prior to the press, which itself did not differ from the baseline likelihood of a glutamate transient in similar epochs without lever pressing during the pre-test period: t_7_ = 2.12, *p *=* *0.07) between 3 and 1 s prior to initiating lever presses (*p* < 0.05). The likelihood of a glutamate transient became elevated again in the 3-s window after the initiating press, which corresponded to the average time at which the next press within a bout occurred (average 2.02 s, sem = 0.12). Given that the average latency between an initiating press and reward delivery was 44.4 s (sem = 10.0; max = 160.6, min = 7.76m), it is unlikely that the glutamate release events that occurred during the 5 s following initiation of reward seeking activity were related to reward receipt (see Supplemental Results and Supplemental Fig. 5 for glutamate transient likelihood around reward receipt). These results corroborate our previous report^21^ and suggest that BLA glutamate release events are increased in frequency and amplitude during instrumental reward seeking, are tightly time-locked to the initiation of instrumental action and positively correlate with instrumental performance.

### BLA glutamate release during Pavlovian-instrumental transfer

**Figure 4:**
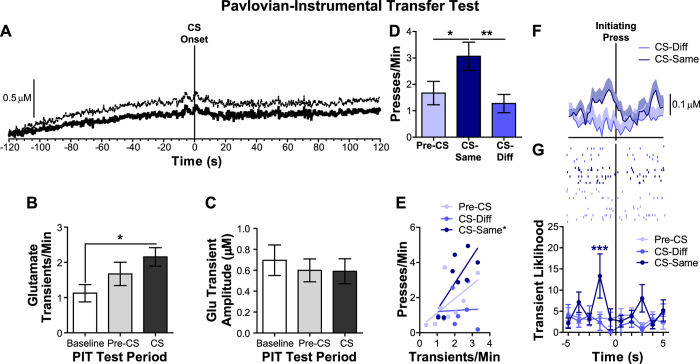
Basolateral amygdala glutamate release during Pavlovian-instrumental transfer. (**A**) Average glutamate concentration change (μM) during the 2-min conditioned stimulus (CS) presentation (beginning at time 0 s) and immediately preceding 2-min pre-CS period averaged across trials during the PIT test for each rat and then averaged across rats. Dashed lines represent the between-subjects +1 SEM. (**B**) Glutamate transients that reached threshold were counted and averaged for each rat across the 2-min pre-test baseline period, the 2-min pre-CS periods and the 2-min CS periods. (**C**) Glutamate transient the amplitude (μM) was calculated as the peak amplitude of the transient minus the baseline glutamate concentration. (**D**) Lever-press rate (Presses/min) averaged across levers during the control Pre-CS periods compared to pressing during CS presentation distinguished by whether it was on lever that, in training, earned the same outcome as predicted by the presented CS (CS-Same Actions) relative to pressing on the opposite lever (CS-Diff Actions). Main effect of CS: F_2,14_ = 8.85, *p *=* *0.003. (**E**) Glutamate transient frequency (Transients/min) v. lever-press rate (Presses/min) between-subjects correlation. (**F**) Glutamate concentration v. time traces for the 5 s prior to and after initiating presses (occurring at time 0 s) during the CS averaged across all initiating presses for a representative subject. Shading reflects +1 SEM across trials. (**G**) The likelihood of a glutamate transient distributed in 1-s bins, 5 s prior to and after initiating presses (occurring at time 0 s; first press after a >6 s pause in pressing). Glutamate transient likelihood is defined as the percentage of initiating presses with a glutamate transient in the represented 1-s time bin. Asterisks represent significance relative to the first 1-s time bin. Raster plot displays corresponding raw data; each subject is represented on an individual line on the y-axis with tick color reflecting trial type. Tick marks represent the peak time of each glutamate transient that reached threshold surrounding initiating presses. Error bars +1 SEM. *p < 0.05, **p < 0.01, ***p < 0.001.

We next measured BLA glutamate concentration changes during PIT to evaluate how BLA glutamate release relates to the cognitive, reward-specific representations generated by reward-predictive cues that allow them to selectively invigorate reward-seeking actions. As can be seen in the group-averaged glutamate concentration v. time trace presented in Fig. 4A, presentation of a Pavlovian CS did not induce any apparent robust or sustained increase in glutamate concentration, although there was a slight overall drift in the baseline current. The reward-predictive cues did, however, elevate the frequency of discrete glutamate release events (Fig. 4B; main effect of Period: F_2,14_ = 4.25, *p *=* *0.04), but the amplitude of these transients was, on average, not significantly altered by CS presentation (Fig. 4C; main effect of Period: F_2,14_ = 1.72, *p *=* *0.22). Glutamate transients were more frequent than the pre-test (no behavior or cues) baseline period during the CS (*p* < 0.05), but not pre-CS period (*p *>* *0.05) in a manner similar to that seen during Pavlovian conditioning (no effect of Extinction: Pavlovian conditioning v. PIT test F_1,5_ = 0.74, *p *=* *0.43 or Extinction x CS interaction: F_2,10_ = 0.11, *p *=* *0.89- see also Supplemental Results and Supplemental Figure 6 for data from the Pavlovian conditioning test). That there was not a significant difference in glutamate transient frequency during the CS relative to the pre-CS period is likely due to the reward-predictive nature of the operant box context because of its pairing with reward during instrumental conditioning. Indeed, rats were exploring the chamber, entering the food-delivery port and lever pressing during this period.

As during instrumental conditioning, BLA glutamate transient frequency correlated with lever pressing, but only during the CS and with only those actions for which performance was selectively invigorated by the CS, *i.e.*, CS-Same actions (see behavioral results in Fig. 4D). During the pre-CS period the positive correlation between glutamate transient frequency and lever pressing was weakened, relative to instrumental conditioning as a result of extinction; there was a positive, but non-significant between-subjects relationship between pre-CS lever-press rate and pre-CS glutamate transient frequency (r_8_ = 0.61, *p *=* *0.11; Fig. 4E). The significant positive correlation reemerged when the CS was present, but only for the CS-Same action (r_8_ = 0.75, *p *=* *0.03), such that those rats for which the CSs caused a stronger selective invigoration of responding it also induced a higher frequency of BLA glutamate transients. Glutamate transient frequency did not significantly correlate with the performance of actions during the CS that, during training, earned an outcome different from that predicted by the CS (CS-Different actions; r_8_ = 0.04, *p *=* *0.92).

More importantly, examination of the representative example in Fig. 4F and raster plot displaying raw glutamate transient peak times for all subjects in Fig. 4G suggests that BLA glutamate transients were time-locked to the initiation of reward-seeking activity (see Table 1) specifically on the CS-Same action. There was an overall effect of CS (Pre-CS v. CS-Same v. CS-Different initiating presses; F_2,14_ = 6.00, *p *=* *0.01) on the likelihood of glutamate transients (normalized to number of initiating pressing) distributed around initiating lever presses, with no significant effect of Time (F_9,63_ = 1.21, *p *=* *0.31) and a marginally insignificant Time x CS interaction (F_18,126_ = 1.50, *p *=* *0.10; Fig. 4G- bottom). The likelihood of a glutamate transient was only significantly elevated during the CS prior to initiating presses on the CS-Same action (1-s bin, 2 s prior to CS-Same initiating presses, *p* < 0.001 relative to the control, 1-s bin 5 s prior to the initiating press). Initiation of CS-Same pressing was significantly more likely than initiation of CS-Different pressing to be preceded (within 5 s) by a glutamate transient (average percentage of CS-Same initiating presses preceded by glutamate transient: 28.87%, sem = 6.09; CS-Different initiating presses: 12.96%, sem = 5.39; t_7_ = 2.78, *p *=* *0.04). See Supplemental Results and Supplemental Figure 7 for an expanded window of analysis for these data. These data suggest that extinction of the press-reward and context-reward associations during the PIT test disrupted the normal temporal relationship between glutamate release and reward seeking, but this was restored when action performance was motivated by outcome-specific information provided by reward-paired cues. See the Supplemental Results and Supplemental Figure 8 for evaluation of the relationship between BLA glutamate transients and Pavlovian conditioned approach during the PIT test.

**Figure 5:**
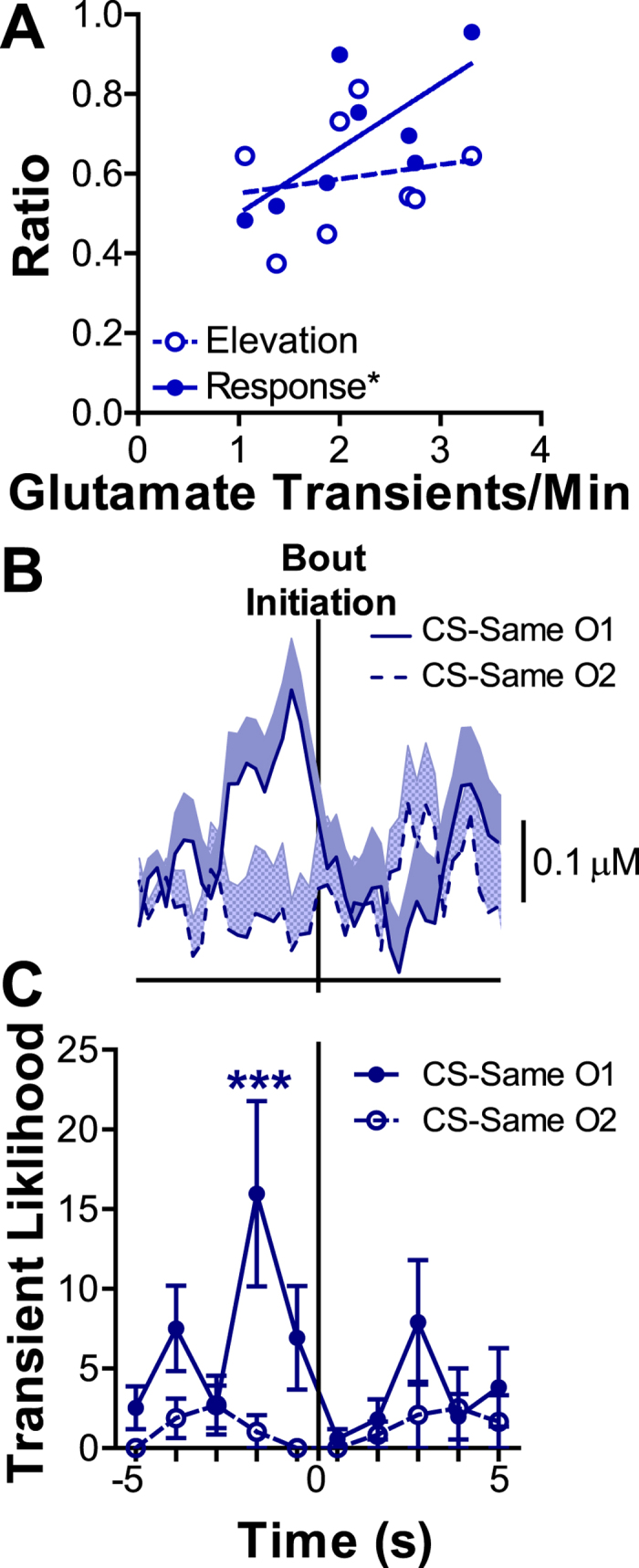
Outcome specificity of basolateral amygdala glutamate release during Pavlovian-instrumental transfer. (**A)** Between-subjects correlation between glutamate transient frequency (Transients/min) and elevation [CS pressing/(CS + Pre-CS pressing)] v. response [CS-Same/(CS-Same + CS-Different)] ratio. (**B**) Glutamate concentration v. time traces for the 5 s prior to and after initiating presses (occurring at time 0 s) was averaged across all CS-Same initiating presses during the PIT test for the same representative subject as in 4F. Traces are divided by the type of outcome the action earned. Shading reflects the +1 SEM across trials. (**C**) The likelihood of a glutamate transient distributed in 1-s bins, 5 s prior to and after CS-Same initiating presses (occurring at time 0 s). Data are divided by the two CS-Same trial types. O1: Outcome 1 is defined as the outcome earned by the action that was exclusively (for 6/8 subjects) or preferentially (for 1/8 subjects) preceded by transient glutamate release. For the single subject in which he glutamate signal did not distinguish between outcome types outcome 1 was randomly assigned to the grain pellet outcome. Likelihood of a glutamate transient is defined as the percentage of initiating presses that had a glutamate transient in the represented 1-s time bin. Asterisks represent significance relative to the control 1-s time bin 5 s prior to the press. *p < 0.05, ***p < 0.001.

Interestingly, on the macro scale glutamate transient frequency positively correlated with the ratio of responding between actions during the CS (r_8_ = 0.71, *p *=* *0.049; Fig. 5A), but did not significantly correlate with all non-discriminate CS responding (r_8_ = 0.22, *p *=* *0.60). This correlation with the CS response ratio was significant even when controlling for overall response rate during instrumental conditioning (partial correlation: r_8_ = 0.81, *p *=* *0.03) or during the pre-CS period (partial correlation: r_8_ = 0.83, *p *=* *0.02) and when controlling for the CSs’ ability to non-discriminately elevate reward seeking (partial correlation: r_8_ = 0.75, *p *=* *0.05). These data suggest that BLA glutamate transients may be related to the motivational influence of outcome-specific representations.

To further support this interpretation we exploited the utility of the two different specific PIT trial types (one for each predicted outcome). If BLA glutamate transients reflect outcome-specific motivational information then, because glutamate biosensors record from BLA microenvironments, recorded glutamate transients for a given subject/recording location should be specific to CS-Same responding for only one outcome type. If however, glutamate transients are simply related to all motivated lever pressing then they should occur prior to CS-Same actions regardless of expected outcome. The data provide evidence in support of the former. For 6/8 subjects glutamate transients were time-locked to initiating presses on the CS-Same action exclusively for only one outcome type (defined as outcome 1). Which outcome served as outcome 1 was not a function of outcome type (pellets v. sucrose), lever, action-outcome arrangement, CS type, outcome preference, or PIT effect magnitude. In the other 2 subjects glutamate transients showed an outcome-selectivity ratio of 0.57 and 0.50, respectively. Fig. 5B displays a representative trial-averaged glutamate concentration v. time trace around initiating presses on the CS-Same action divided by each outcome type. As is clear from this figure, glutamate concentration increased prior to initiating presses on the CS-Same action, but only for one outcome type. The data averaged across subjects support this observation. There was a main effect of Outcome type (F_1,7_ = 6.54, *p *=* *0.04), and of Time (F_9,63_ = 2.48, *p *=* *0.02), as well as a Time x Outcome type interaction (F_9,63_ = 2.27, *p *=* *0.03) on the likelihood of a glutamate transient in the 10-s period around initiating presses on the CS-Same action. Together these results suggest that BLA glutamate transients encode outcome-specific motivational information provided by reward-predictive cues.

## Discussion

The data collected here indicate that transient fluctuations in BLA glutamate release are time-locked to and correlate with instrumental reward seeking and that during PIT these glutamate transients are time-locked to and correlate with only those actions invigorated by outcome-specific motivational information provided by a reward-predictive stimulus. This correlational relationship was bolstered by evidence that blockade of AMPA, but not NMDA iGluRs attenuates the selective invigorating influence of reward-predictive stimuli over reward seeking.

That transient BLA glutamate release events were related to instrumental reward seeking replicates previous results demonstrating a similar relationship^21^ and extends this to show that BLA glutamate transients were associated with the actions that initiated reward seeking following reward delivery or a pause in activity, rather than actions occurring within a bout of reward seeking. This release may drive the previously-reported increases in BLA cell body activity that occur prior to instrumental action^38^,^39^ and have been hypothesized to encode outcome expectations^38^. The results here suggest that BLA glutamate input may encode information vital for motivating goal-directed action, because after a task is well-learned such information is only necessary for initial actions within a chunk^40^,^41^,^42^. Indeed, these results corroborate evidence that amygdala neurons in primates show prospective activity that reflects internally-generated plans towards future goals^43^. Of course BLA glutamate release is not exclusively related to instrumental action; glutamate transients were more likely to occur around instrumental actions, but release events were detected throughout the instrumental test, including especially large events during reward receipt.

The temporal relationship between BLA glutamate transients and reward-seeking was tight, but it was not one-to-one and glutamate release events that reached the detection threshold occurred at a rate lower than might be expected for the major excitatory neurotransmitter and primary input signal to the amygdala. The recording technique employed here measures changes in extrasynaptic glutamate concentration, which, because these signals are abolished by tetrodotoxin^21^, are a proxy measure for the tightly-regulated^44^ synaptic overspill^44^,^45^. Glutamate release within the synapse might, therefore, be expected to relate to a much larger percentage of, if not all, initiating lever presses.

This study provided a novel evaluation of the profile of BLA glutamate release during appetitive Pavlovian conditioning. Although the baseline drift in electrochemical measurements do not allow for a definitive answer, the data showed no indication that Pavlovian reward-predictive cues elicited an overall elevation in glutamatergic tone, contrary to what might be expected if BLA glutamate signaling conveys a sustained, cue-induced, emotional valence or motivational signal. This finding is interesting in light of the wealth of data from single-unit electrophysiological recordings showing that reward-predictive stimuli increase BLA cell body firing^35^,^36^,^38^,^46^,^47^. Single-unit recordings are biased towards monitoring mostly from output neurons and the glutamate release recorded here reflects input and local activity, but it is this glutamate input from thalamic^46^,^48^,^49^ or cortical afferents^6^ that is thought to drive the cell body excitation. There are three potential explanations for this discrepancy. First, for the reasons mentioned above it is possible that glutamate biosensors do not provide the adequate sensitivity to measure glutamate that is being released to drive BLA activity upon CS presentation. Secondly, the aforementioned CS-induced BLA neural activity may not be driven by glutamate. We find this unlikely given data demonstrating strengthened glutamatergic thalamic-BLA synapses during Pavlovian conditioning^46^, but dopamine release has been shown to directly excite BLA projection neurons^50^. Thirdly, key task differences may explain the discrepancy between the current glutamate input recordings and the previously reported cue-evoked cell body firing. In the previous reports BLA cell body firing was robustly elicited by short-duration (2–5 s) CSs that predicted immediate reward with strong certainty. The long-duration (2-min) CS probabilistically paired with reward in our task provides more a context for reward and may not induce a robust increase in BLA cell body firing. In support of this, preliminary evidence suggests that a longer duration (30-s) CS that predicts reward at a variable latency is more likely to induce an inhibition in BLA cell body firing^51^, which corroborates our current glutamate release results. Clearly, further interrogation of both glutamate release and cell body activity in similar Pavlovian tasks is necessary. Such investigation may lead to important information regarding potential differences between excitatory BLA input and output activity and of the role of such signaling in Pavlovian reward prediction.

Importantly, although there was no detected sustained CS-induced increase, glutamate release did show transient elevations during the PIT test. Following extinction of the response-reward (and context-reward) association the relationship between BLA glutamate transients and instrumental reward seeking was degraded. During the CS, however, this relationship was restored; glutamate transients were significantly more likely to occur time-locked to reward-seeking activity selectively on the action that shared the same rewarding outcome as the CS. That this relationship was restored despite the fact that the CS induced only a modest (relative to the pre-CS period) increase in the frequency of glutamate transients suggests it was not merely a coincidence of both elevated responding (which the analysis controlled for) and elevated glutamate transients. These data lend support to the hypothesis that BLA glutamate transients encode the outcome-specific motivational information provided by reward-predictive stimuli. In further support of this, cue-induced transient glutamate release only correlated with the ratio of responding during the CS, which is thought to reflect the CS’s cognitive, outcome-specific motivational influence. This is accords with the relationship between BLA activity and biasing influence of cues over instrumental action in humans^52^. Moreover, for each subject/recording location glutamate transients encoded only one specific outcome type. Because biosensors record glutamate in BLA microenvironments, the presumption is that for each subject glutamate input signals related to the other outcome were released in a microenvironment outside the sampling space. In the one subject for which glutamate release did not show outcome specificity the biosensor was likely receiving intermixed glutamate input for both outcome types. This specificity in the glutamate input signal suggests that rather than relating simply to motivated lever pressing, the BLA glutamate release detected here encoded outcome-specific information.

BLA glutamate release events were shown to relate to the outcome-specific motivational influence of Pavlovian stimuli, but it is unlikely that these signals participate in the decision-making process itself. If the BLA, and glutamate release therein, was required for the decision process then blockade of BLA AMPA receptors should have not only attenuated actions on the lever that earned the same outcome as the CS, but also increased responding on the alternate lever, indicating an inability to select between actions on the basis of the CS-provided outcome expectation. Instead, BLA AMPA receptor inactivation (Experiment 1) and BLA lesions^11^,^13^ only attenuate the selective invigorating influence of CSs. Lesions to either the orbitofrontal cortex or mediodorsal thalamus do, however, cause such non-discriminate CS-induced response invigoration^11^,^53^. Both of these regions send excitatory projections to the BLA^7^,^8^,^9^ and unilateral, ipsilateral orbitofrontal cortex inactivation abolishes reward seeking-related BLA glutamate transients^21^. Therefore, the glutamate signals detected here likely arise directly from the orbitofrontal cortex, or, given that our sensor placements are located primarily in the basal amygdala, indirectly from this region via projections from the lateral amygdala^54^. BLA glutamate release may, therefore, be vital for invigorating the performance of actions planned in the orbitofrontal cortex by incorporating outcome-specific motivational value. Indeed, the BLA is vital for outcome-specific representations of motivationally significant, but not valueless events^55^. Correlates of the latter have, however, been identified in the orbitofrontal cortex^56^. This interpretation is also supported by the myriad data suggesting the BLA is required for other behaviors that rely on outcome-specific value information^10^,^57^,^58^,^59^,^60^ and evidence that BLA neural activity can be outcome specific^33^,^36^,^38^,^47^ and may encode such value in the rodent^33^,^38^,^61^, primate^36^,^62^,^63^,^64^, and even human BLA^65^.

In summary, the findings here support a role for rapid BLA glutamate signaling in the motivating influence of outcome-specific representations, in this case provided by a Pavlovian reward-predictive stimulus. These results lay the groundwork for further exploration of the role of BLA excitatory glutamatergic signaling and modulation of such signaling in the variety of reward-seeking behaviors that require retrieval of reward-specific information and are relevant to understanding the neuropsychological disorders marked by a disruption in such cognitive processing.

## Methods

### Subjects

Male Long Evans rats (Experiment 1: n = 17, Experiment 2: n = 8; Charles River Laboratories, Wilmington, MA) weighing between 280–340 g were group housed and were handled for 3–5 days prior to training. Training and testing took place during the dark phase of the 12:12 h reverse dark:light cycle. Rats were maintained on a food-deprived schedule whereby they received 10–12 g of their maintenance diet daily in order to maintain 85% free-feeding body weight. All procedures were conducted in accordance with the NIH Guide for the Care and use of Laboratory Animals and approved by the UCLA Institutional Animal Care and Use Committee.

### Apparatus

Training took place in 16 Med Associates operant boxes (East Fairfield, VT). The chambers contained 2 retractable levers that could be inserted to the left and right of a recessed food delivery port in the front wall. A photobeam entry detector was positioned at the entry to the food port. The chambers were also equipped with syringe pump to deliver 20% sucrose solution in 0.1 ml increments through a stainless steel tube into a custom-designed well in the food port and a pellet dispenser to deliver a single 45-mg grain pellet (Bio-Serv, Frenchtown, NJ). Both a tone and white noise generator were attached to individual speakers on the wall opposite the lever and magazine. A 3-watt, 24-volt house light mounted on the top of the back wall opposite the food cup provided illumination. For experiment 2 all testing was conducted in a single Med Associates operant box housed within a continuously-connected, copper mesh-lined sound attenuating chamber and outfitted with an electrical swivel (Crist Instrument Co, Hagerstown, MD) connecting a headstage tether that extended within the operant chamber to the potentiostat recording unit (Fast-16 mkIII, Quanteon, LLC, Lexington, KY) positioned outside the operant chamber.

### Behavioral training and surgery

Rats were first given Pavlovian training (see below and Fig. 1A), to associate two distinct 2-min duration auditory stimuli (CSs) each with a different palatable food reward, delivered into a single food port on a random-time 30 s schedule. Rats were then separately trained to instrumentally earn these same food rewards by responding on independent levers on a random ratio (RR) 20 schedule (see below). During training the CSs and levers were never presented together. Following training rats underwent surgery using standard aseptic stereotaxic procedures described previously^66^. In Experiment 1 rats were implanted with guide cannula (22-gauge stainless steel, Plastics One, Roanoke, VA) targeted bilaterally 1 mm above the BLA. In Experiment 2 rats were implanted with a unilateral pre-calibrated glutamate biosensor (see below) into the BLA (AP −3.0 mm, ML +5.1, DV −8.0) and a Ag/AgCl reference electrode (200 μm diameter) into the contralateral cortex (histological verification of placements shown in Figs 1B and 2A). Following surgery rats were individually housed and allowed to recover.

#### Pavlovian Training

Rats received 1 Pavlovian training session/day for 8 days. Each session consisted of 8 tone and 8 white noise (75 db, 2-min duration) presentations, during which either 20% sucrose solution or grain pellets, as appropriate, were delivered on a 30-s random-time schedule into a single food-delivery port, resulting in an average of 4 stimulus-outcome pairings per trial (CS-reward relationship counterbalanced). CSs were delivered in pseudo-random order with no more than 2 successive presentations of the same CS and a variable inter-trial interval between 2–4 min (3-min mean). The rate at which rats entered the food port was recorded for the 2-min pre-CS period, for the CS-probe period (interval between CS onset and first US delivery) and for the CS-reward period (interval after first US delivery to CS offset). No levers were present during these Pavlovian conditioning sessions.

#### Instrumental Training

Rats were then given 11 days of instrumental training, receiving 2 separate training sessions per day, one with the lever to the left of the food-delivery port and one with the right lever. Each action was reinforced with a different outcome- either grain pellets or sucrose solution (counterbalanced with respect to the Pavlovian training contingencies). Each session was terminated after 30 outcomes had been earned or after 30 min had elapsed. Actions were continuously reinforced (CRF) for the first day of instrumental conditioning, on days 2-3 rewards were delivered on a random ratio (RR)-2 schedule, increasing to RR-5 for days 4-5, RR-10 for days 6–7 and ending with RR-20 for days 8-11. Importantly, neither CS was present during this instrumental training. The rate of responding on each lever was measured throughout training.

### Experiment 1

#### Outcome-specific Pavlovian- instrumental transfer tests

After 5 days of recovery, rats were given 2 retraining sessions for each instrumental association (2 sessions/day for 2 days) and then one Pavlovian retraining session (see Supplemental Results for performance on these sessions). On the day prior to each PIT test rats were given a single 30-min extinction session where both levers were available, but pressing was not reinforced to establish a low level of responding.

Rats were split into two drug groups, one (n = 8) group receiving bilateral infusions of 0, 1 or 3 μg/side of the selective α-amino-3-hydroxy-5-methyl-4-isoxazolepropionic acid (AMPA)/kainate receptor antagonist NBQX into the BLA and another (n = 9) receiving 0, 1 or 3 μg/side of the selective N-methyl-D-aspartate (NMDA) receptor antagonist AP5 10 min prior to the onset of the PIT test. Each rats was given 3 total PIT tests to allow within-subject drug dose comparisons (test order counterbalanced). During this test both levers were continuously present, but pressing was not reinforced. After 10 min of extinction to re-establish a low level of responding each 2-min CS was presented separately 4 times each in pseudorandom order, separated by a fixed 4-min inter-trial interval. No rewards were delivered during CS presentation. The 2-min prior to each CS presentation served as the ‘pre-CS’ control period. Rats were retrained as above between each PIT test.

#### Drug Administration

AP5 (D-(-)-2-Amino-5-phosphonopentanoic acid) and NBQX (2,3-Dioxo-6-nitro-1,2,3,4-tetrahydrobenzo[*f*]quinoxaline-7-sulfonamide disodium salt) were obtained from Tocris Bioscience (Bristol, UK) and were dissolved in sterile saline vehicle. Drugs were infused bilaterally into the BLA in a volume of 0.5 μl over 1 min via an injector inserted into the guide cannula fabricated to protrude 1 mm ventral to the tip using a microinfusion pump. Injectors were left in place for at least 1 additional min to ensure full infusion. Rats were placed in the operant chamber for the PIT test 10 min after infusion to allow sufficient time for the drug to become effective. The dose range for each drug (1 or 3 μg/side for both AP5 and NBQX) was selected based on previous research showing intra-BLA infusion of these doses to be effective in a variety of Pavlovian and reward-related tasks^67^,^68^,^69^. Drug test order was counterbalanced across rats. On 2 of the retraining days prior to the first PIT test rats were given mock infusions to habituate them to the infusion procedures; injectors were inserted into the cannula and the pump was turned out, but no fluid was infused.

### Experiment 2

#### Electroenzymatic glutamate sensing

Microelectrode array (MEA) probes were fabricated in the Nanoelectronics Research Facility at UCLA and modified for glutamate detection as described previously^17^,^21^. Briefly, these biosensors use glutamate oxidase as the biological recognition element and rely on electro-oxidation, via constant-potential amperometry (0.7 V versus a Ag/AgCl reference electrode), of enzymatically-generated hydrogen peroxide (reporter molecule) to provide a current signal. This current output is recorded and converted to glutamate concentration using a calibration factor determined *in vitro*. Interference from both electroactive anions and cations is effectively excluded from the amperometric recordings, while still maintaining a <1-s response time, by application of Nafion and polypyrrole (PPY) films to the electrode sites prior to enzyme immobilization^17^. Additionally, incorporation of a non-enzyme-coated sentinel electrode on the MEA enabled removal of correlated noise from the glutamate sensing electrode output by signal subtraction (see Data Analysis), as described previously^21^. The average *in vivo* limit of glutamate detection of the sensors used in this study was 0.38 μM (sem = 0.04, range 0.2–0.7 μM), which is an improvement over our previous reports^17^,^21^ and allowed detection of lower amplitude glutamate release events.

#### Reagents

Nafion (5 wt.% solution in lower aliphatic alcohols/H_2_O mix), bovine serum albumin (BSA, min 96%), glutaraldehyde (25% in water), pyrrole (98%), L-glutamic acid, L-ascorbic acid, 3-hydroxytyramine (dopamine) were purchased from Aldrich Chemical Co. (Milwaukee, WI, USA). L-Glutamate oxidase (GluOx) from *Streptomyces* Sp. X119-6, with a rated activity of 24.9 units per mg protein (U mg^−1^, Lowry’s method), produced by Yamasa Corporation (Chiba, Japan), was purchased from US Biological (Massachusetts MA). Phosphate buffered saline (PBS) was composed of 50 mM Na_2_HPO_4_ with 100 mM NaCl (pH 7.4). Ultrapure water generated using a Millipore Milli-Q Water System (resistivity = 18 MΩ cm) was used for preparation of all solutions used in this work.

#### Instrumentation

Electrochemical preparation of the sensors was performed using a Versatile Multichannel Potentiostat (model VMP3) equipped with the ‘p’ low current option and low current N’ stat box (Bio-Logic USA, LLC, Knoxville, TN). *In vitro* and *in vivo* measurements were conducted using a low-noise multichannel Fast-16 mkIII potentiostat (Quanteon), with reference electrodes consisting of a glass-enclosed Ag/AgCl wire in 3 M NaCl solution (Bioanalytical Systems, Inc., West Lafayette, IN) or a 200 μm diameter Ag/AgCl wire, respectively. All potentials are reported versus the Ag/AgCl reference electrode.

#### In Vitro Biosensor Characterization

All biosensors were calibrated *in vitro* to test for sensitivity and selectivity of glutamate measurement. A constant potential of 0.7 V was applied to the working electrodes against a Ag/AgCl reference electrode in 40 mL of stirred PBS at pH 7.4 and 37 °C within a Faraday cage. Data were collected at 80 kHz and averaged over 1-s intervals. After the current detected at the electrodes equilibrated to baseline (approx. 30–45 min), aliquots of glutamate were added to the beaker to reach final glutamate concentrations in the range 5–60 μM. A calibration factor based on analysis of these data was calculated for each electrode. Additionally, aliquots of ascorbic acid (250 μM final concentration) and dopamine (5–10 μM final concentration) were added to the beaker as representative examples of readily oxidizable potential anionic and cationic interferent neurochemicals, respectively, to confirm selectivity for glutamate. For the sensors used in these studies no current changes above the level of the noise were detected to the addition of cationic (dopamine) or anionic (ascorbic acid) interferents, as reported previously^17^,^21^. In order to assess the sensitivity and response time to peroxide at sites uncoated with enzyme, aliquots of H_2_O_2_ (10 μM) were also added to the beaker. Importantly, electrodes coated with PPy, Nafion and BSA/glutaraldehyde, but not GluOx, showed no detectable response to glutamate, despite being sensitive to H_2_O_2_. Indeed, there was less than a 10% statistically insignificant (t_8_ = 1.28, p = 0.24) difference in the H_2_O_2_ sensitivity on control electrode sites relative to enzyme-coated sites, indicating that any changes detected *in vivo* on the enzyme coated sites could not be attributed to endogenous H_2_O_2_.

#### Online, near-real time glutamate detection during reward seeking

Prior to the start of each test session (see Fig. 2B) rats were placed in the recording operant chamber and the biosensor was tethered to the potentiostat via the electrical swivel for application of the 0.7 V potential. Oxidative current was recorded at 80 kHz and averaged over 0.25-s intervals. The recorded amperometric signal was allowed to stabilize prior to session onset (approx. 45 min). The 2-min period immediately prior to the onset of the behavioral session was used as the baseline period for comparison of glutamate concentration changes in the absence of any reward-related behavior to those induced by responding during test. On the first test day rats received a single Pavlovian conditioning test that was identical to Pavlovian training described above. On the second day of testing rats were given instrumental conditioning sessions as described above, but with the ratio requirement progressively increasing from fixed ratio-1 to RR-20 after each 5^th^ outcome earned. On the last test day rats received a single PIT test, identical to that described for Experiment 1.

### Data Analysis

Unless otherwise mentioned, all data were processed using Microsoft Excel (Redmond, WA), then compiled and statistically analyzed with GraphPad Prism (La Jolla, CA) and SPSS (IBM Corp, Chicago, IL). For all hypothesis tests, the α level for significance was set to *p* < 0.05. The data were analyzed with paired t-tests, repeated-measures ANOVAs (with post hoc analysis correcting for multiple comparisons), correlation and regression, where appropriate.

#### Behavioral Analysis

Lever-press rate was the primary behavioral output measure. During the PIT test lever pressing for the 2-min pre-CS period was compared to that during the CS period, which was divided for presses on the lever that, during training, earned the same outcome as the cue predicted (*i.e.*, CS-Same presses) versus those on the other available lever (*i.e.*, CS-Different presses).

Because rats tended to organize their instrumental lever-pressing activity into clusters of many lever presses in quick succession, we focused on those presses that initiated reward seeking (*i.e*., ‘initiating presses’) excluding presses that occurred within a pressing bout when analyzing the temporal relationship between glutamate release and instrumental activity. For the instrumental conditioning test an ‘initiating press’ (23.68% of total presses, sem = 1.77) was defined as the first press after collection of an earned reward or, because rats often disengaged from the lever and then reinitiated reward seeking, the first press after >6 s pause in pressing. Similar definitions of initiation of reward seeking and instrumental bouts defined by pauses in activity have been described previously^70^,^71^,^72^. The average reward receipt to next initiating press latency was 23.10 s (sem = 7.47) for the instrumental tests, indicating that those glutamate release events that occurred within 5 s prior to initiating presses were related to the performance of this action and not to consumption of the previously earned reward. For the instrumental test we compared the temporal relationship between glutamate and these initiating presses to that with all presses (including both initiating and intra-bout presses) in order to determine if BLA glutamate release was related to individual actions or initiation of reward-seeking activity. For the 2 instrumental conditioning tests data were collapsed across sessions, because there was neither a significant main effect of Earned outcome type (F_1,7_ = 0.14, *p *=* *0.72), nor Outcome type x Time interaction (F_9,63_ = 0.86, *p *=* *0.56) on the temporal relationship between glutamate and instrumental activity. Because no rewards were delivered during the PIT test an initiating press was defined as the first press after a pause in pressing of >6 s. On average 57.00% (sem = 9.30) of all presses during the pre-CS period were initiating presses, with 40.03% (sem = 6.82) of CS-Same presses and 46.06% (sem = 6.45) of CS-Different presses considered initiating presses.

#### Neurochemical Recording Analysis

Analysis details and characterization of glutamate release events have been described previously^21^. Each electrode’s baseline current was subtracted from its current output after equilibration (average current over 10-s period, 2 min prior to test onset). The current changes from baseline on the PPY/Nafion-coated sentinel electrode were subtracted from current changes on the PPY/Nafion/GluOx glutamate biosensor electrode to remove noise correlated among the electrodes on the MEA. The glutamate biosensor response then was converted to glutamate concentration using an electrode-specific calibration factor obtained *in vitro*, which averaged 152.86 μM/nA.

Because the baseline current detected at the glutamate sensing electrodes drifted over minutes during the course of the test session (see examples in Fig. 3A), as is typical for electrochemical recordings, we focused our analysis on transient glutamate concentration changes. Mini Analysis (Synaptosoft, Decatur, GA) was used to determine the frequency, and amplitude of these rapid changes. A fluctuation in the glutamate trace was deemed a glutamate transient if it was at >2.5x the RMS noise sampled from the pre-test baseline period. To determine the transient amplitude a baseline was taken by averaging 3 sample bins around the first minima located 0.5–5 s before the peak and this baseline was subtracted from the peak amplitude. If one peak followed another within 5 s the baseline was taken after the first peak to distinguish these events. Peaks with a total duration below 0.5 s or with an immediately preceding or following negative deflection greater than half the peak amplitude were considered noise spikes and were omitted from the analysis. The frequency and amplitude of glutamate transients were averaged across the 2-min pre-test baseline period and compared to that averaged across the instrumental conditioning test, or that averaged across the 2-min pre-CS or 2-min CS periods for the PIT test. These variables were correlated against instrumental press rate during each test.

We also evaluated the precise temporal relationship between transient glutamate release events and behavioral output by assessing the likelihood of a glutamate transient in the time immediately surrounding a lever press. The likelihood of a glutamate transient was defined as the percentage of lever presses that had a glutamate transient in each of 10, 1-s time bins distributed evenly around the event. This time window was selected to ensure release events in the 5 s prior to an initiating presses were specifically related to that particular initiating press and not to contaminating events (e.g., reward delivery during the instrumental test, termination of a previous bout, head entries into the food-delivery port etc.). During the instrumental test the average reward collection to next initiating press latency was 23.10 s (SEM = 7.47), and by definition an initiation press must have been preceded by a >6 s pause in pressing. Values were normalized as a percentage of presses, as opposed to number or percentage of transients, to control for the variation in pressing that resulted from experimental manipulation (*e.g.*, CS presentation), which could confounded interpretation. In all cases the 1-s time bin 5 s prior to the event served as the control bin for statistical comparison because in this time bin glutamate transient likelihood did not differ from the baseline likelihood of a glutamate transient in similar epochs without lever pressing during the pre-test period (t_7_ = 2.12, *p *=* *0.07). Raw glutamate transient peak times for each subject in the 5 s prior to and after initiating press are displayed in the raster plots shown in Figs 3G and 4G.

### Histology

At the conclusion of each experiment rats were anesthetized with Nembutal and trans-cardially perfused with 0.9% saline followed by 10% formalin saline. The brains were removed and post-fixed in formalin, then cryosectioned into 60 μm slices, mounted onto slides and stained with cresyl violet. Light microscopy was used to examine sensor or cannula placement in the BLA. Histological data are presented in Fig. 1B (Experiment 1) and 2A (Experiment 2). Four rats were removed from Experiment 1 due to cannula misplacement or bilateral BLA tissue damage.

## Additional Information

**How to cite this article**: Malvaez, M. *et al*. Basolateral amygdala rapid glutamate release encodes an outcome-specific representation vital for reward-predictive cues to selectively invigorate reward-seeking actions. *Sci. Rep*. **5**, 12511; doi: 10.1038/srep12511 (2015).

## Supplementary Material

Supplementary Information

## Figures and Tables

**Table 1:** Pavlovian-instrumental transfer test lever pressing bouts.

	Pre-CS	CS: Same	CS: Diff
Total Bouts	11.63	17.125*	10.38
	*3.50*	*3.60*	*2.95*
Average Presses/Bout	2.69	3.77	2.12
	*0.38*	*1.31*	*0.18*
Average Bout Duration (s)	3.61	5.03	2.58
	*0.54*	*1.73*	*0.51*
% Presses = Bout Initiating	57.00	40.03	46.06
	*9.93*	*6.82*	*6.45*

As in the instrumental conditioning test, rats organized their reward seeking in bouts of lever pressing during the PIT test. Because no rewards were delivered during this test an initiating press was defined as the first press after a pause in pressing of >6 s. Pressing bout information is presented in this table. Numbers represent average values, with values in italics below representing the between-subject SEM. During the PIT test rats showed significantly more lever-pressing bouts on the CS- Same action relative to both the CS-Different action and to the pre-CS period (*p < 0.05, in both cases; main effect of CS period F_2,4_ = 5.81, *p *=* *0.01). Main effects for all other measures were non-significant.
