# Corrigendum: Basolateral amygdala rapid glutamate release encodes an outcome-specific representation vital for reward-predictive cues to selectively invigorate reward-seeking actions

**DOI:** 10.1038/srep20891

**Published:** 2016-02-15

**Authors:** Melissa Malvaez, Venuz Y. Greenfield, Alice S. Wang, Allison M. Yorita, Lili Feng, Kay E. Linker, Harold G. Monbouquette, Kate M. Wassum

Scientific Reports 5: Article number: 12511; 10.1038/srep12511 published online: 07272015; updated: 02152016

The original version of this Article contained an error in the Abstract.

“These data the hypothesis that transient glutamate release in the BLA can encode the outcome-specific motivational information provided by reward-predictive stimuli”.

now reads:

“These data support the hypothesis that transient glutamate release in the BLA can encode the outcome-specific motivational information provided by reward-predictive stimuli” 

This has now been corrected in the PDF and HTML versions of the Article.

