# Supplementary material for: Basolateral amygdala rapid glutamate release encodes an outcome-specific representation vital for reward-predictive cues to selectively invigorate reward-seeking actions
Source: Sci Rep. 2015 Jul 27;5:12511. doi: 10.1038/srep12511 (PMC4648450; doi:10.1038/srep12511)
Supplement: Supplementary Information [file srep12511-s1.pdf]

Basolateral amygdala rapid glutamate release encodes an outcome-specific representation vital for reward-predictive cues to selectively invigorate reward-seeking actions

Melissa Malvaez[1], Venuz Y. Greenfield[1], Alice S. Wang[1], Allison M. Yorita[2], Lili Feng[2], Kay E. Linker[1] and Harold G. Monbouquette[2], Kate M. Wassum[1,3]

1. Dept. of Psychology, UCLA, Los Angeles, CA 90095. 2. Dept. of Chemical Engineering, UCLA, Los Angeles, CA 90095, USA. 3. Brain Research Institute, UCLA, Los Angeles, CA 90095, USA.

Supplemental Material:
Supplemental Results
Supplemental Figures 8

Supplemental Results

Experiment 1

Pre-Test training results

After BLA cannulae implantation and recovery rats in Experiment 1 were retrained on both the Pavlovian and instrumental contingencies. During the post-operative Pavlovian retraining session rats entered the food-delivery port significantly more during the CS probe period (AMPA Group: average entry rate 25.84, sem=2.60; NMDA Group: 25.41, sem=3.37) than during the pre-CS period (AMPA Group: 12.37, sem=1.82; NMDA Group: 10.36, sem=1.77); there was only a main effect of CS ($F_{1,15}$=136.90, p<0.001), with neither an effect of Future group (AMPA v. NMDA; $F_{1,15}$=0.13, p=0.72), nor Group x CS interaction ($F_{1,15}$=0.43, p=0.52). Similarly, there was no pre-existing group difference in instrumental response rate (t_{15}=0.13, p=0.90; AMPA Group average press rate: 46.60, sem=3.77; NMDA Group average press rate: 47.42, sem=4.78).

Neither AMPA nor NMDA receptor activation is required for Pavlovian conditioned food-port approach responding

After both intra-BLA AMPA and NMDA receptor blockade rats were able to show Pavlovian conditioned food-port approach responding (Supplemental Figure 2). For the AMPA group (Supplemental Figure 2A) there was a significant main effect of CS (pre-CS v. CS; $F_{1,7}$=76.26, p<0.0001) on food-port entry rate, with a marginally insignificant main effect of NBQX dose ($F_{2,14}$=3.56, p=0.06) and NBQX dose x CS interaction ($F_{2,14}$=3.43, p=0.06). Despite the marginal drug effect, approach to the food-delivery port was significantly elevated during the CS relative to the pre-CS period after intra-BLA infusion of vehicle (p<0.001), low dose NBQX (p<0.05) and high dose NBQX (p<0.001). Similarly, for the NMDA Group (Supplemental Figure 2B) there was an overall significant main effect of CS (pre-CS v. CS; $F_{1,8}$=27.36, p<0.001), with a marginally insignificant main effect of AP5 dose ($F_{2,16}$=2.89, p=0.08) and no AP5 dose x CS interaction ($F_{2,16}$=0.81, p=0.46). For this group the CS elevated food-port approach relative to the pre-CS

period after intra-BLA infusion of vehicle ($p<0.001$), low dose AP5 ($p<0.05$) and high dose AP5 ($p<0.05$).

That the effect of AMPA receptor blockade was primarily detected in the influence of the reward-predictive cues over instrumental activity (see main text and Figure 1C), rather than Pavlovian-conditioned responding does not necessarily suggest that BLA glutamate signaling is unrelated to conditioned food-port approach responding. Indeed, if such responding were guided by a specific reward representation we would expect it to require AMPA receptor signaling much like the specific PIT effect. In this task both reward types were collected from a shared food port such that conditioned food-port approach did not require reward-specific information. This may explain why food port approach was less affected by AMPA receptor blockade. Future studies that specifically assay the information used to guide conditioned food port approach responding are required to determine the contribution of BLA AMPA receptor to Pavlovian conditioned responding.

Experiment 2

BLA glutamate release during instrumental conditioning- extended analysis.

To include a broader time window depicting glutamate transient fluctuations surrounding reward seeking activity, we evaluated glutamate fluctuations in the 8 s prior to and 10 s after a lever press. This window was chosen to provide a longer pre-press time frame for a broader view of the baseline and to encompass after the press the average reward-seeking bout duration of 8.20 s (sem=1.33). For this analysis we again compared glutamate transient likelihood around those presses that initiated reward seeking (*i.e.,* ‘initiating presses’, excluding presses that occurred within a pressing bout) to that around to all lever presses (including both initiating and intra-bout presses). These data are presented in Supplemental Figure 4. Supplemental Figure 4A shows the representative trial-averaged glutamate concentration v. time trace. We counted glutamate transients in 8, 1-s bins prior to and 10, 1-s bins after each press. These raw data for each subject

are displayed in the raster plot (Supplemental Figure 4B- top). Statistical analysis of the data collapsed over 1-s intervals (to match biosensor response time) and averaged across subjects (Supplemental Figure 4B- bottom) found significant effect of Time surrounding the press ($F_{17,119}$=1.90, *p*=0.022), a significant effect of Type of press (Initiating press v. All presses, $F_{1,7}$=65.55, *p*<0.0001) with no significant Time x Type of press interaction ($F_{17,119}$=1.15, *p*=0.32). As with the results presented in the main text, BLA glutamate transients tended to occur before initiating lever presses. The likelihood of a glutamate transient was elevated (relative to the control 1-s time bin, 8 s prior to the bout) between 3 and 1 s prior to an initiating press (*p*<0.01-0.05). We also evaluated transient fluctuations in glutamate around only those presses that initiated a bout of 2 or more lever presses to examine the relationship between glutamate release and specifically the initiation of reward-seeking *bouts*. These data are shown in Supplemental Figure 4C. As is clear from this figure the likelihood of a glutamate release event was also similarly elevated in the seconds prior to actions that initiated a bout of reward-seeking activity. The increased variability in transient likelihood in these expanded time window analyses may be attributed to contamination by additional behavior events.

We also evaluated transient fluctuations in glutamate surrounding receipt of the earned reward during the instrumental conditioning test. As can be seen in the representative trial-averaged glutamate concentration v. time trace in Supplemental Figure 5A or the group glutamate transient likelihood data presented in Supplemental Figure 5B, reward delivery produced a slight, but non-significant increase in glutamate transient likelihood following receipt of the earned reward (no significant overall main effect of time: $F_{4.24, 29.69}$=1.85, *p*=0.14, all post-hoc comparisons *p*>0.05). Given that the average reward receipt to next initiating press latency was 23.10 s (sem=7.47), it is unlikely that these glutamate release events that occurred immediately following reward receipt contaminated the 5-s analysis window that preceded the initiating press.

BLA glutamate release during Pavlovian conditioning.

On the first test day rats received a single Pavlovian conditioning test that was identical to Pavlovian training described in the main text methods. These data are presented in Supplemental Figure 6. Two rats were eliminated from the Pavlovian analysis due to unstable amperometric signals during only this test day. These rats had RMS noise levels on the Pavlovian conditioning test that were greater than 2x the standard deviation of the average noise level (mean RMS Noise with these rats included = 0.168 µM, sem= 0.061). On the instrumental and PIT test days the noise level for these rats was less than 1 standard deviation away from the mean RMS noise and their data was included in these analyses.

As can be seen in the representative example in Supplemental Figure 6A or the data averaged across trials and across rats presented in Supplemental Figure 6B, although there was an overall drift in the baseline current, presentation of a Pavlovian CS did not induce any apparent robust or sustained increase in glutamate concentration. The reward-predictive cues did, however, induce a moderate elevation in the frequency of discrete glutamate release events (Supplemental Figure 6C; main effect of Period: $F_{2,10}$=8.99, p=0.006, baseline v. CS p<0.01, baseline v. pre-CS p>0.05), but the amplitude of these transients was, on average, not significantly altered by the CS (Supplemental Figure 6D; no main effect of Period: $F_{2,10}$=2.21, p=0.16). That there was not a significant difference in glutamate transient frequency during the CS relative to the pre-CS period during this test is likely due to the reward-predictive nature of the operant box context because of its pairing with reward during instrumental conditioning. Indeed, rats were exploring the chamber and entering the food-delivery port during this period (see Supplemental Figure 6E).

The reward-predictive cues were also effective in elevating food-port approach responding. There was a significant main effect of CS on food-port entries ($F_{2,10}$=7.51, p=0.01) with entries being elevated during the CS-Probe (during the interval between CS onset and first reward delivery; p<0.05) and CS-Reward (interval after first reward delivery to CS offset; p<0.05) periods relative to the pre-CS period. Glutamate frequency during each period did not significantly correlate with the performance of this Pavlovian conditioned approach behavior between-subjects

during the pre-CS (r_6=0.15, p=0.78), CS-Probe (r_6=0.09, p=0.87), or CS-Reward (r_6=0.28, p=0.58) periods (Supplemental Figure 6F). Interestingly however, the likelihood of a glutamate transient did appear to be elevated during this checking behavior (i.e. *after* the food-port entry- see Supplemental Figure 6A) and this was especially prominent when a reward was found. During CS periods, we compared the likelihood of glutamate transients around food-port entries in which there was a reward present versus those in which there was not (normalized to total entries for each condition). There were significant main effects of Time surrounding the CS food-port entry ($F_{9,45}$=2.48, p=0.02) and Reward presence ($F_{1,5}$=15.28, p=0.01) on glutamate transient likelihood, with no significant interaction between these variables ($F_{9,45}$=0.99, p=0.47; Supplemental Figure 6G). Food-port entries were more likely to be followed by a glutamate transient than preceded; the likelihood of a glutamate transient was elevated 1 s after the food-port entry regardless of whether a reward was present (p<0.05, in both cases) and this remained elevated 2 s after food-port entry when a reward was present (p<0.05). Entries during the CS in which a reward was found in the food-port were more likely to be followed by a glutamate transient than entries in which there was not a reward (average percentage of CS entries with reward present followed by transient: 32.56, sem=8.84, without a reward present: 15.25%, sem=5.06; t_5=2.99, p=0.03). These data suggest that BLA glutamate release events are related to a process that occurs when reward-paired cues are present and when subjects are checking for and consuming rewards during the CS. Indeed, when the rat checks the food port for reward during the CS he gains information that promotes new learning, such that these BLA glutamate signals may be linked to the expectation of reward and the encoding of this reward-specific information into the stimulus-outcome relationship. This exciting hypothesis warrants further investigation.

BLA glutamate release during Pavlovian-instrumental transfer- extended analyses.

As can be seen in the representative average glutamate concentration v. time trace presented in Supplemental Figure 7A or the data averaged across trials and across rats presented in

Supplemental Figure 7B with an expanded time frame surrounding initiation of instrumental activity, transient BLA glutamate release was time-locked to the initiation of reward-seeking activity on the CS-Same actions. There was an overall effect of CS (Pre-CS v. CS-Same v. CS-Different initiating presses; $F_{2,14}$=3.74, *p*=0.05) on the likelihood of glutamate transients (normalized to number of presses) distributed around initiating presses, with no significant effect of Time ($F_{1,7}$,119=1.23 *p*=0.25) and an insignificant Time x CS interaction ($F_{34,238}$=1.23, *p*=0.19). The likelihood of a glutamate transient was only significantly elevated during the CS prior to initiating presses on the CS-Same action (1-s bin, 2 s prior to CS-Same bout, *p*=0.05 relative to the control, 1-s bin 8 s prior to the bout).

Relationship between BLA glutamate release events and Pavlovian conditioned food-port approach responding during the PIT test.

During the PIT test presentation of the reward-predictive CSs resulted in an increase in Pavlovian conditioned approach responding (main effect of CS on food-port entries: t_7=5.45, *p*=0.001). As with the Pavlovian conditioning test, the frequency of glutamate release events did not significantly correlate with food-port entry rate during either the pre-CS (r_8=0.31, *p*=0.40) or CS (r_8=0.35, *p*=0.46) period (Supplemental Figure 8B). However, when evaluating the distribution of glutamate transients surrounding food-port entries during the PIT test there was a significant effect of Time ($F_{9,63}$=2.88, *p*=0.006) with neither effect of CS (pre-CS v. CS; $F_{1,7}$=0.13, *p*=0.73), nor interaction between these factors ($F_{9,63}$=0.86, *p*=0.56; Supplemental Figure 8C). During the 1-s bin before and after a food-port entry the likelihood of a glutamate transients was significantly elevated for both the pre-CS (*p*<0.001, in both cases) and CS (1 s pre: *p*<0.001, 1 s post: *p*<0.05) periods, suggesting a tight temporal relationship between glutamate release events and food-port checking. That the likelihood of a glutamate transient was elevated prior to the food-port entry during the PIT test, which was not the case during the Pavlovian conditioning test, is likely

because entries tended to follow CS-Same lever press actions which were themselves preceded by glutamate transients.

Supplemental Figures

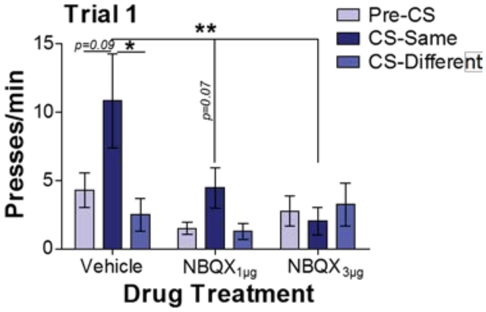

Supplemental Figure 1: Basolateral amygdala AMPA receptor inactivation on the first trial of the Pavlovian-instrumental transfer test. Lever press rate (presses/min) averaged across levers during the control Pre-CS period compared to pressing on lever that, in training, earned the same outcome as predicted by the CS presentation (CS-Same) relative to pressing on the opposite lever (CS-Different) for the AMPA antagonist group. Because in the trial-averaged data shown in Figure 1C the pre-CS response rate was low (~2 presses/min) it is possible that the lack of a significant effect of the AMPA receptor antagonist could be attributed to a floor effect. To evaluate this we focused on the first trial of the PIT test in which the pre-CS response rate was significantly higher. The effect of AMPA blockade is identical to that detected on the trial-averaged data. There was an overall main effect of CS period ($F_{2,14}$=9.39, p=0.003), a marginally insignificant effect of NBQX Drug ($F_{2,14}$=3.49, p=0.06), and a marginally significant interaction between these factors ($F_{4,28}$=2.23, p=0.09). CS-Same responding was lower following intra-BLA NBQX infusion than vehicle control (high dose: p<0.01, low dose: p=0.07). Intra-BLA NBQX did not significantly alter pre-CS baseline response rates (p>0.05, in both cases) during this trial. These results confirm that the effect of AMPA receptor blockade was specific to the selective invigorating influence of cues over action performance. Error bars $\pm$ 1 SEM. *p<0.05, **p<0.01.

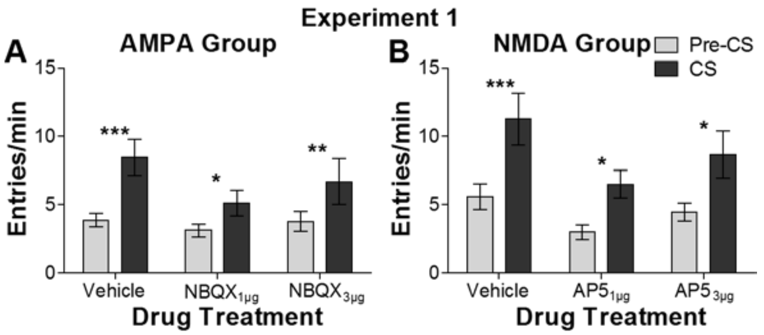

Supplemental Figure 2: Pavlovian conditioned food-port approach responding during the Pavlovian-instrumental transfer test under AMPA or NMDA receptor blockade. A. Food-port head entry rate (entries/min) during the control Pre-CS periods compared the CS period for the AMPA (**A**) or NMDA (**B**) group. Error bars + 1 SEM. *p<0.05, **p<0.01, ***p<0.001.

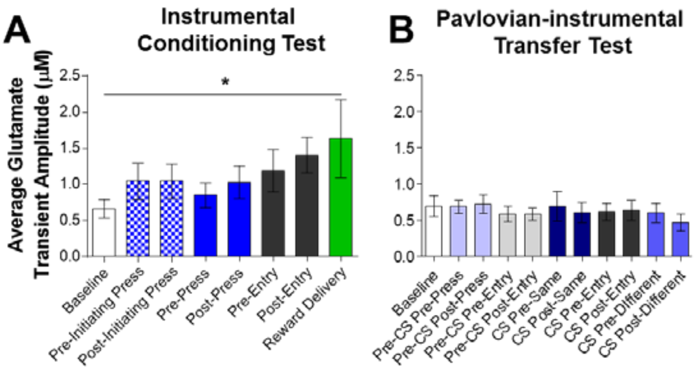

Supplemental Figure 3: Amplitude of behavioral event-related basolateral amygdala glutamate release. A. Amplitude of BLA glutamate transients during the pre-test baseline compared to press-, food-port entry- and reward-related glutamate transients during the instrumental conditioning test. There was a significant main effect of event on the amplitude of glutamate transients ($F_{5,35}$=2.66, p=0.04), with reward-delivery associated glutamate release events being the largest in amplitude and significantly larger than pre-session baseline glutamate release events (p<0.05- controlling for multiple comparisons). When evaluating only the amplitude of pre-press glutamate release events relative to baseline there was a significant main effect of Event Type ($F_{2,23}$=6.17, p=0.01), with the amplitude of glutamate transients prior to initiating presses (p<0.05), but not all (p>0.05) lever presses being significantly higher than baseline glutamate transient amplitude. **B.** Amplitude of glutamate release events during the pre-test baseline compared to press- and food-port entry-related glutamate transients during the outcome-selective Pavlovian-instrumental transfer test. Data are divided by the pre-CS control and CS presentation. These data could not be statistically analyzed because some events (Pre-CS and CS-Different presses) never had a time-locked glutamate release event in this test. Error bars indicate ± 1 SEM. *p<0.05.

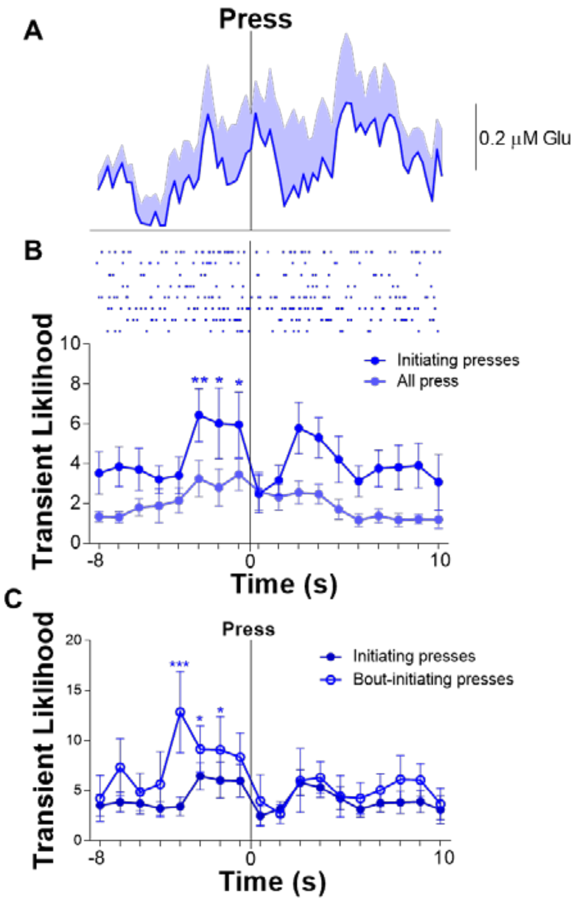

Supplemental Figure 4. Transient BLA glutamate release events around reward-seeking during instrumental conditioning- expanded analysis window. A. Glutamate concentration v. time trace for the 8 s prior to and 10 s after the initiation of reward seeking (occurring at time 0 s) during the instrumental test averaged across all initiating presses for a representative subject (same subject as main text Figure 3F). Shading reflects +1 SEM across trials. **B.** The likelihood of a glutamate transient distributed in 1-s bins, 8 s prior to and 10 s after initiating presses (first press after earned reward or a $\geq$6 s pause in pressing) v. all presses combined (intra-bout presses and bout-initiating presses). The press occurs at time 0 s. Glutamate transient likelihood is defined as the percentage of presses with a glutamate transient in the represented 1-s time bin. Raster plot displays corresponding raw data for initiating presses; each subject is represented on an individual line on the y-axis. Tick marks represent the peak time of each glutamate transient that

reached threshold surrounding bout-initiating presses. Asterisks represent significance relative to the control 1-s time bin 8 s prior to the press. **C.** The likelihood of a glutamate transient distributed in 1-s bins, 8 s prior to and 10 s after initiating presses (same as above) v. those presses that initiated a bout of 2 or more lever presses. Error bars $\pm$ 1 SEM. *$p<0.05$, **$p<0.01$, ***$p<0.001$.

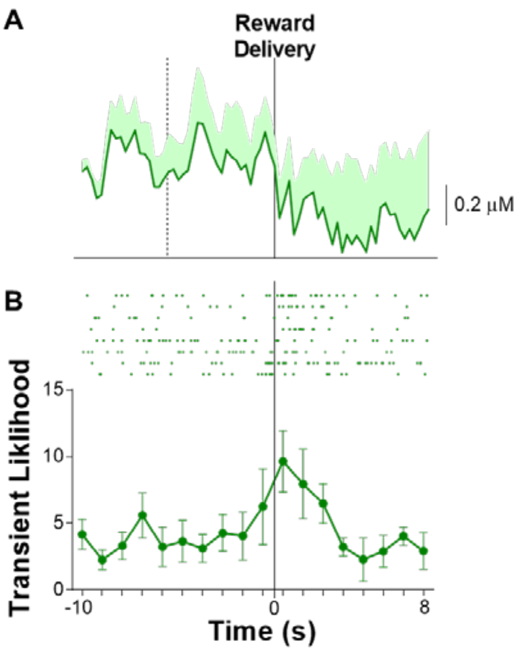

Supplemental Figure 5. Transient BLA glutamate release events around earned reward delivery during instrumental conditioning. A. Glutamate concentration v. time trace for the 10 s prior to and 8 s after delivery of the earned reward (occurring at time 0 s) during the instrumental test for a representative subject (same subject as shown in Figure 3F and Supplemental Figure 4A). Shading reflects +1 SEM across trials. The dashed line indicates the average time of the preceding initiating press for this specific subject. **B.** The likelihood of a glutamate transient distributed in 1-s bins, 10 s prior to and 8 s after reward delivery (occurring at time 0 s). Glutamate transient likelihood is defined as the percentage of reward deliveries with a glutamate transient in the represented 1-s time bin. Raster plot displays corresponding raw data; each subject is represented on an individual line on the y-axis. Tick marks represent the peak time of each glutamate transient that reached threshold surrounding reward delivery. Asterisks represent significance relative to the control 1-s time bin, 10 s prior to the reward delivery. Error bars $\pm$ 1 SEM.

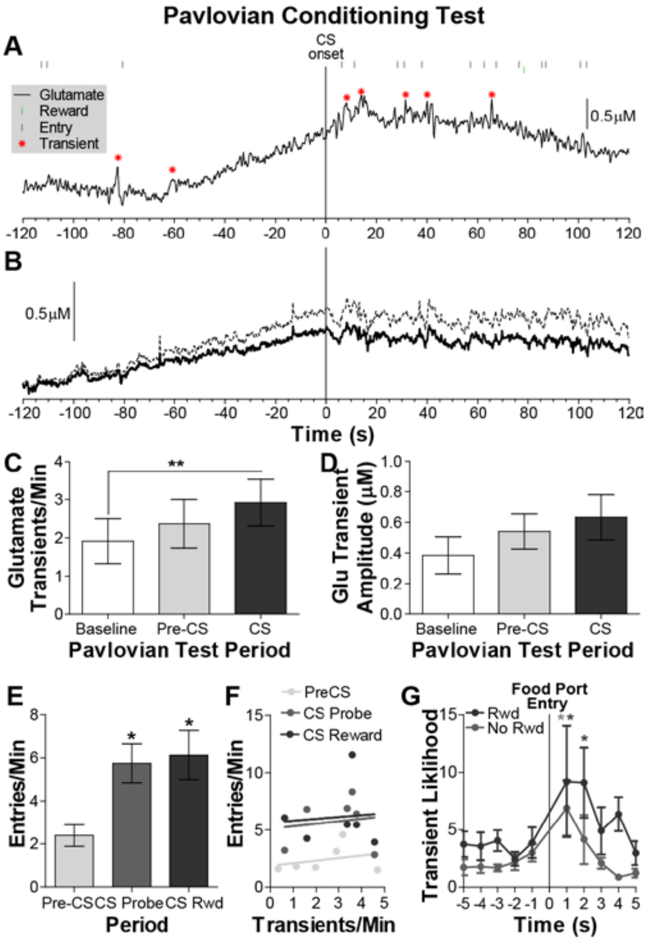

Supplemental Figure 6: Transient BLA glutamate release events during Pavlovian conditioning. A. Representative glutamate concentration v. time trace during a single Pre-CS and CS period. CS onset occurred at time 0 s. Asterisks represent significant transient glutamate concentration fluctuations above baseline (Transients). Food-delivery port entries are marked as lines above the trace. **B.** Average glutamate concentration change (μM) during the 2-min conditioned stimulus presentation (CS) presentation and the immediately preceding 2-min pre-CS periods averaged across trials for each rat and averaged across rats. Dashed lines represent +1 SEM. **C.** BLA glutamate transient events that reached threshold were counted for the entire test session and then averaged for each rat across the 2-min pre-test baseline period prior to the behavioral session onset (Baseline), the 2-min pre-CS periods and the 2-min CS periods. **D.** For each glutamate transient the amplitude (μM) was calculated as the peak amplitude of the transient

minus the baseline glutamate concentration (first minima 0.5-5 s prior to the peak). This was averaged for each rat across the 2-min pre-test baseline period prior to the session onset (Baseline), the 2-min pre-CS periods and the 2-min CS periods. **E.** Head entries into the food-delivery port averaged across the 2-min no-cue (Pre-CS) periods, during the time at which the CS was presented, but prior to the first reward delivery (CS-Probe period) and during the CS after rewards were delivered (CS-Reward). **F.** Non-significant between-subjects correlation between glutamate transient frequency (Transients/min) and food-port entry rate (Entries/min). **G.** The likelihood of a glutamate transient distributed in 10, 1-s bins, evenly around food-port entries during the CS separated for those CS entries in which a reward was (Rwd, dark gray) or was not (No Rwd, light gray) present. The food-port entry occurs at time 0 s. Likelihood of a glutamate transient is defined as the percentage of entries that had a glutamate transient in the represented 1-s time bin. Asterisks represent significance relative to the control 1-s time bin 5 s prior to the entry. Error bars $\pm$ 1 SEM. *$p<0.05$, **$p<0.01$, ***$p<0.001$.

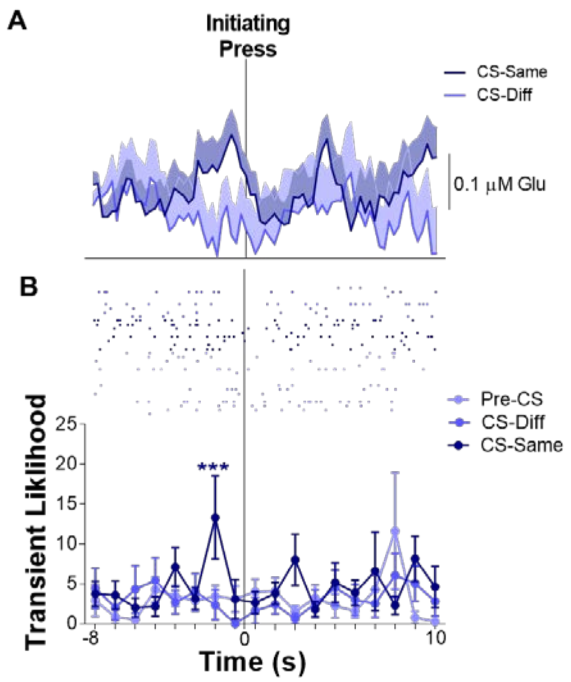

Supplemental Figure 7: Transient BLA glutamate release events around reward-seeking during Pavlovian-instrumental transfer- expanded analysis window. A. Glutamate concentration v. time traces for the 8 s prior to and 10s after initiating lever presses (occurring at time 0 s) during the CS averaged across all initiating presses for a representative subject (same subject as in main text Figure 4F). Shading reflects +1 SEM across trials. Initiating presses defined as the first press after a $\geq$6 s pause in pressing. **B.** The likelihood of a glutamate transient distributed in 1-s bins, 8 s prior to and 10 s after initiating presses (occurring at time 0 s). Glutamate transient likelihood is defined as the percentage of initiating presses with a glutamate transient in the represented 1-s time bin. Raster plot displays corresponding raw data; each subject is represented on an individual line on the y-axis with tick color reflecting trial type. Tick marks represent the peak time of each glutamate transient that reached threshold surrounding initiating presses. Asterisks represent significance relative to the control 1-s time bin 8 s prior to the press. Error bars $\pm$ 1 SEM. ****p*<0.001.

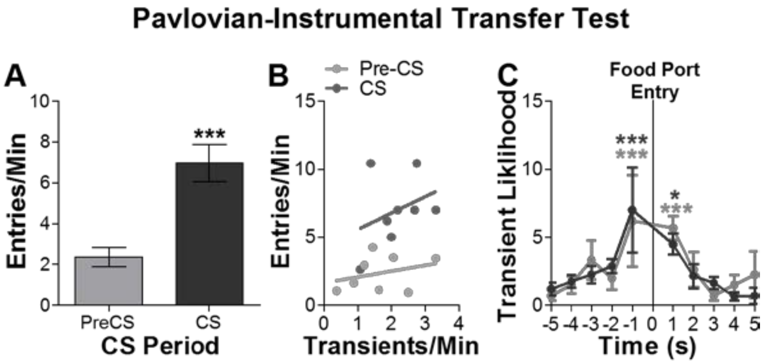

Supplemental Figure 8: Relationship between BLA glutamate release and Pavlovian conditioned food-port approach responding during the Pavlovian-instrumental transfer test. A. Head entries into the food-delivery port averaged across the 2-min no-cue (Pre-CS) and CS periods. **B.** Non-significant between-subjects correlation between glutamate transient frequency (Transients/min) and food-port entry rate (Entries/min) during the Pre-CS and CS periods. **C.** The likelihood of a glutamate transient distributed in 10, 1-s bins evenly around food-port entries during the pre-CS and CS periods. The food-port entry occurs at time 0 s. Likelihood of a glutamate transient is defined as the percentage of entries that had a glutamate transient in the represented 1-s time bin. Asterisks represent significance relative to the control 1-s time bin 5 s prior to the entry. Error bars $\pm$ 1 SEM. ***p<0.001.
